# Foreign Summary

**Published:** 1839-02-09

**Authors:** 


### FOREIGN SUMMARY.

*On the Treatment of Gonorrhœa.* By Professor Graves.—I do not know any practical point on which greater diversity of opinion exists, than the administration of injections in gonorrhœa. In Dublin, students are generally taught that their use is improper and dangerous. The following are the chief objections to which they are said to be liable:—1st. They do not diminish the urethral inflammation, though they dry up the discharge, and consequently they lay the foundation for stricture, or more immediately occasion the inflammation to descend along the urethra, until it extends to the membranous portion, the prostate, or even the bladder. 2dly. Their use renders swelled testicle and sympathetic bubo more frequent. 3dly. It is argued that the use of any measures, except such as are purely antiphlogistic, must be improper in a disease accompanied by so many indubitable signs of inflammation. Let us closely examine this last objection, and we shall find it to possess more apparent than real weight, for analogy proves that the principle on which it depends is by no means universally applicable, particularly in cases of specific inflammation. When surgeons placed their sole reliance on antiphlogistic measures, local or general, in the treatment of purulent ophthalmia, the results were truly disastrous; and however exhausted the patient became from excessive bleeding by the lancet and leeches, aided by large and frequently-repeated doses of tartar emetic internally, the local inflammation proceeded in its rapid and destructive course, scarcely influenced, never effectually checked, by the treatment adopted. I have seen a man treated with bleeding, general and local, employed I might say to excess, and aided by rapid and profuse mercurial salivation,—I have seen, in the patient referred to, both eyes destroyed by purulent ophthalmia in a few days. Not long ago I was called during the night to visit a young gentleman in a hotel; he had gonorrhœa, and went to bed without any complaint of the eyes, but was soon wakened by pain in the left eye. It was evidently purulent ophthalmia, and was cured in the course of a few hours by relays of leeches, and a strong sulphate of zinc collyrium, carefully applied. After thousands had lost their vision from the effects of this disease, it was at length discovered that some who adopted a totally different mode of practice, and who treated the purulent ophthalmia in its very commencement with strong astringent and corrosive applications, were eminently successful. This led many army surgeons, more especially Mr. Guthrie, to investigate the subject with care. You are aware of the important practical results at which he arrived, and of the great improvement which has consequently taken place in ophthalmic surgery, leading to the application of solid nitrate of silver, or its concentrated solution, of sulphate of copper, &c. &c., to the mucous membrane of the eye in the first stages of purulent ophthalmia—a mode of treatment which our predecessors would not have hesitated to pronounce most hazardous and destructive.

With respect to the objection that the treatment of gonorrhœa by injection lays the foundation for strictures, I beg most distinctly to deny the truth of the assertion: whatever diminishes the intensity, and shortens the duration of the urethral inflammation, must tend to diminish, and not to increase, the liability of strictures. Compare the violence and duration of a gonorrhœa skilfully treated from its very beginning, by injections, with a case where no injections are employed—the physician's reliance being exclusively placed on perfect rest, confinement, fasting, and cooling medicines; compare two such patients—observe how the one is perfectly cured of his disease in a few days, without confinement, and without any deviation from his usual diet and habits (I speak now of two cases coming under treatment in a day or two after the appearance of the very first symptoms;) and then watch the other through sufferings protracted week after week, until his constitution is debilitated by confinement and low diet: how often do we find the discharge from the urethra increasing daily, in spite of the general and local antiphlogistic remedies employed, until it is profuse in the extreme, and accompanied by great ardor urinæ, painful erections, irritation of the bladder, and chordee. Now I will fearlessly assert that a medical man who gets the care of *a recent gonorrhœa in a healthy constitution*, is grievously to blame if he permits this series of bad symptoms to supervene. I do not deny that these symptoms will at length give way to the antiphlogistic treatment, leeches along the perineum, stupes, inunction of the skin covering the urethra, with mercurial ointment and belladonna, &c. &c. These remedies will in the end get rid of the disease, but then at what a loss of time and strength! I again repeat the assertion, and I do it emphatically, that a gonorrhœa treated by injections *from the beginning*, can generally, in persons of sound constitution, be cured in a few days. When a gonorrhœa has been allowed to continue several weeks, it often so alters the vitality, and probably the structure of the affected tissues, that a cure is uncertain, and frequently the treatment becomes both perplexing and tedious; when a gleet supervenes, then remedies even the most judiciously selected frequently fail altogether: these facts prove the necessity of curing the disease, in every instance, as soon as possible.

But, gentlemen, we must here enter into details, and first as to the manner of injecting the urethra. Many believe that the inflammation produced by the specific poison of gonorrhœa is seated chiefly, if not exclusively, in the portion of the urethra near the orifice; and hence they are only anxious to introduce the injected fluid a short distance in that canal. Nothing can be more unfounded than this opinion, and nothing more injurious than the practice to which it gives rise. The inflammation which gonorrhœa produces in the urethra is by no means confined to the third of the canal near its orifice, but even in recent cases it extends much farther, and it cannot therefore be efficiently treated by injections, which do not come into contact with the whole extent of the inflamed surface. Unless you yourselves teach your patients how to inject, not one in ten of them will do it properly. Of this, an extensive experience has convinced me. Over and over again have I been told that there was no use in trying injections in a particular case, as they had been already tried in vain; and on accurately inquiring into the patient's mode of injecting, the result has been the discovery that he was quite ignorant of the proper method. The pewter syringe or squirt used must be in proper order, so as to work easily with the pressure of one finger; otherwise when the end is in the urethra, and the patient tries to inject the fluid contained in the syringe, the point is very apt to be hitched against the urethra, in consequence of the force thus suddenly applied. The point of the syringe must be carefully introduced at least half an inch within the lips of the urethra, and the fore finger and thumb of the left hand must then be so applied as to press the lips of the urethra gently on the syringe, so as effectually to prevent the reflux and consequent escape through the orifice, of the injected fluid. When the fluid is thrown in, the patient will feel it in the urethra, which it will gently distend as far down as the membranous portion, if a sufficient quantity be injected. Some persons have an idle fear about the ill consequences which would arise were any of the injection to arrive at the bladder. An ordinary pewter syringe does not contain more than a drachm and a half, which is about the quantity required for one injection. When the fluid has been injected, the point of the syringe is to be withdrawn, and the lips of the urethra kept closed with the finger and thumb, for at least two minutes, when, the pressure being removed, the injected fluid will be thrown out from the urethra with considerable force, in consequence of the elasticity of that canal. These directions, gentlemen, are by no means unnecessary; indeed, I never treat a patient without seeing that he knows how to inject, for I find that many say they know the right method, who are quite ignorant of it, and who, consequently, do themselves more harm than good by making the attempt.

It is not my object to enter at present into the especial therapeutics of gonorrhœa, and consequently it would be foreign to my plan to speak of the various substances which may be used in injections; for an account of these I must refer to authors who have written at large on this subject. As a general rule, you ought to commence with weak solutions of the astringents you prefer, which solutions may be used five or six times a day, and may be daily increased in strength. *An injection should seldom be used so strong as to cause, at the time, any thing like severe pain of the urethra.* In this respect we must not closely imitate the example of *eye-waters*, such as that used by the Egyptian *hakim.* I have, indeed, often known very strong injections used at the first trial, and which, though they produced great pain for many minutes after their introduction, yet were very effectual in rapidly curing the disease, and that without any bad consequence. (This is more especially the case with nitrate of siver, which, although a powerful remedy, I have found unmanageable, and therefore not to be recommended.) Still, however, by far the safer and most prudent practice is to commence with astringent injections, so weak that, when used, they may produce merely a sense of titillation, or of very inconsiderable smarting. It is often difficult at first to hit off, if I may use the expression, the precise strength required; and therefore I always give my patients particular instructions, and desire them, if the injection is at all too irritating, to dilute it with water to the desired degree of strength. The sensibility of the urethra diminishes very rapidly when an injection of proper strength is applied to the inflamed surface, so that the solution may be daily rendered more astringent. I have told you that astringent injections are suited to every case of gonorrhœa at the commencement of the disease, and that, when properly used during the first, second, or third day, they almost always cut it short. It is not so when the disease has attained its *acmé*, and the inflammation is at its height, accompanied by profuse discharge, chordee, &c. &c. Even then, however, injections properly managed will tend to assist the local antiphlogistic measures; but in such cases we must always commence by using mere mucilaginous warm water, and must add the astringents at first very sparingly, and must increase their proportions vary cautiously. I omitted to observe, *that always, before using an injection, the patient ought to clear the urethra by voiding a little urine.* Such directions, gentlemen, may appear to many prolix and unnecessarily minute; but not knowing any author who has condescended to give accurate accounts respecting these matters, I have thought it my duty to lay them before you, being convinced of their utility.

Before I conclude, it is right to put you on your guard about the mischief which may ensue if you attempt to prescribe astringent injections during the secondary or inflammatory stage of gonorrhœa, without previously having ordered such general and local antiphlogistic treatment as is required to diminish the existing inflammation; nor will even this be sufficient to ensure success, unless you take care that your patient remains quietly at home for a few days. and observes a spare vegetable diet. A person who will not follow your directions in these matters, cannot use astringent injections during this stage of the disease with benefit, or even impunity. In the first stage, and in the third, it is not absolutely necessary to enjoin rest and abstinence; it is, indeed, better and more prudent that the patient should remain in his room, and should observe low diet for a day or two; but in some cases this is impracticable, and then he must, as far as possible, avoid stimulant food and much walking exercise.—*London Medical Gazette.*

---

*On the Treatment of Hydrocele, by the injection of the Tincture of Iodine.* By Dr. Openheim.—An interesting report by Dr. Openheim, on the treatment of hydrocele by the injection of the tincture of iodine into the tunica vaginalis, has been translated for the Dublin Journal of Medical Science, from a German periodical. Dr. O.'s preference for the iodine over the injections, is decided. From the cases he has communicated, we have before us the favourable results of the injections of iodine, both in children and grown up persons of every age, in large and small hydroceles, when the disease was acute, and when chronic; when occurring on one or both sides, both in cases where the operation was undertaken for the first time, and in those in which puncturation had often been performed; nay, even in one case, where the wine injection had in vain been employed by a skilful hand; lastly, in pure, simple, hydrocele, as well as when complicated with hæmatocele. (Hydro hæmatocele.)

In none of the cases treated by me, he says, was the reaction so violent as to require recourse to any internal or external remedies. The greater number of my patients were able to pursue their wonted employments without injury in three or four days, many even in shorter time than this, and some on the very day on which the operation had been performed. In three to four weeks, and at latest after six weeks, every trace of disease had disappeared from those who had been operated on. Many were perfectly cured in a fortnight.

These favourable results have determined me in all cases in private practice, where I have no reason to suspect a disease of the testis, or its coverings, to have recourse to the iodine injection, and to reserve the previously practised operation of incision, or laying open the sac, for those cases where probably the hydrocele is complicated with a degeneration, or some other disease of the testis, or of the tunica vaginalis.

It is easy to be explained, how different observers have arrived at very contradictory results in the application of this remedy, some considering it useless, on account of the trifling reaction caused by it; whilst others, on the contrary, thought it acted too violently, so as to cause suppuration and gangrene of the scrotum. The tincture of iodine is very differently prepared in different pharmacopœias; in some there is more, and in others less of the iodine dissolved, (the Hamburgh as well as the Prussian, says, forty-eight grains of iodine to one ounce of rectified spirit; the Parisian only forty-grains. By the less degree of diffusibility of the iodine in water, the iodine contained in the tincture is very soon precipitated when mixed with water, and it is the more the longer it may remain, so that at last there is nothing but a mixture of water and alcohol. A preparation which unites in such different proportions, cannot be expected to produce similar effects; I find it then most expedient to mix the ingredients at the time of using them, and to make the degree of pain suffered by the patient, the criterion for leaving the injection a longer or shorter time in the tunica vaginalis. The mixture may also be applied warm, for then the iodine will remain longer united with the spirit. By the employment of the caoutchouc flask in place of the simple syringe, there is a double advantage to be derived, the permitting of the fluid to pass many times back and forwards, without any danger of extravasation into the cellular tissue, and by shutting out the light to prevent the separation of the iodine.

---

*Carbonic Acid.*—It is a decided fact that carbonic acid is one of the few remedies which are advantageous in all kinds of phthisis, whether mucous, suppurating, or scrofulous, and which improve and lessen the expectoration, and make it easier, while they diminish the hectic fever; nay, this remedy has sometimes even produced a perfect cure. I appeal to my own experience as well as that of others, on the effects of Seltzer and similar simple carbonated waters.—*London Medical Gazette, from Hufeland: Praktische Uebersicht der vorzüglichsten Heilquellen Teutschlands.*

---

*The Influenza at Lisbon.* By Dr. Lima Leitao.—In the beginning of February the report spread in Lisbon, that the influenza, which had been raging for several weeks in England, was on board several English ships lying at anchor in the Tagus. On the 20th of February the first cases occurred in the hospital, and at the same time the disease showed itself in the town, and attacked whole families. The reporter regrets that he can give no exact meteorological observations, having no instruments; he merely remembers in general, that until the end of December the winter was not severe; but that the first days of January were very cold, and on the 2d it snowed more than it had ever done in the memory of man, so that Lisbon, to the astonishment of its inhabitants, had the look of a town in the north of Europe. From the 10th of January, however, till the end of February, the sun shone warmly, although the wind blew strong and cold. He makes three degrees of the disease:—

*First degree.*—The usual symptoms of the first attack are followed by a burning in the throat, some hoarseness and dry cough, pain in the anterior part of the head, want of appetite, and thirst. This form of the disease lasted from four to six days, with mild evening exacerbations, the cough becoming moist, and nightly perspiration setting in.

*Second degree.*—In this form the well-known catarrhal symptoms were all incomparably more severe and obstinate, the cough more violent and more dry, with oppression of the chest and dyspnœa. The symptoms gradually grew milder, and the attack lasted from five to eight days.

*Third degree.*—The chest symptoms were not so prominent, but there was more affection of the head, amounting in some cases to lethargy. The duration of the disease in this form was the same.

*General treatment.*—This consisted of warm foot-baths, emollient clysters, and mild sudorifics, such as infusions of elder and poppy flowers; with soothing diet, and orange-water for drink. Only in cases where the oppression of the chest was very great, or the affection of the head and the somnolence very violent, from ten to thirty ounces of blood were taken away at three times. Too vigorous a treatment with emetics, purgatives, and strong diaphoretics, and unseasonable bleeding prolonged the disease.—*Lond. Med. Gaz., from Zeitschrift für die gesammte Medicin,* for October 1838, *from the Journal da Sociedade das Sciencias Medicas de Lisboa.*

